# Supplementary material for: Intra-Tumour Heterogeneity Is One of the Main Sources of Inter-Observer Variation in Scoring Stromal Tumour Infiltrating Lymphocytes in Triple Negative Breast Cancer
Source: Cancers (Basel). 2021 Aug 31;13(17):4410. doi: 10.3390/cancers13174410 (PMC8431498; doi:10.3390/cancers13174410)
Supplement: Supplementary file 1 [file cancers-13-04410-s001.zip › cancers-1348780-supplementary.pdf]

Supplementary Materials: Intra-Tumour Heterogeneity is One of the Main Sources of Inter-Observer Variation in Scoring Stromal Tumour Infiltrating Lymphocytes in Triple Negative Breast Cancer

Darren Kilmartin, Mark O'Loughlin, Xavier Andreu, Zsuzsanna Bagó-Horváth, Simonetta Bianchi, Ewa Chmielik, Gábor Cserni, Paulo Figueiredo, Giuseppe Floris, Maria Pia Foschini, Anikó Kovács, Päivi Heikkilä, Janina Kulka, Anne-Vibeke Laenkholm, Inta Liepniece-Karele, Caterina Marchiò, Elena Provenzano, Peter Regitnig, Angelika Reiner, Aleš Ryška, Anna Sapino, Elisabeth Specht Stovgaard, Cecily Quinn, Vasiliki Zolota, Mark Webber, Davood Roshan, Sharon A. Glynn and Grace Callagy

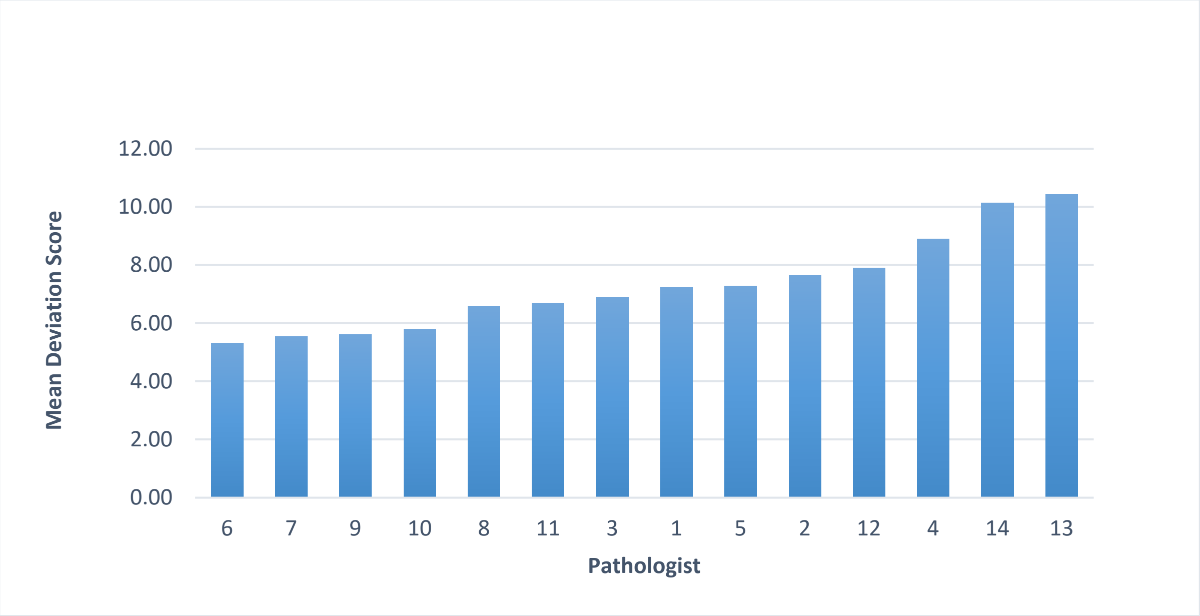

Figure S1. Deviation of each pathologist's score from the mean sTILs score for FOV in circulation 2. The deviation of each pathologist's raw sTILs score from the mean sTIL score from all pathologists for each FOV ($n = 147$) in circulation 2 was calculated. The deviation was then averaged across all FOV to generate a mean deviation score for each pathologist across all 147 FOV. The mean deviation score was 7.28 (range 5.32–10.43). From the graph, two pathologists (numbers 14 and 13) appeared as outliers compared to other pathologists with higher deviation scores and with the greatest deviation (+2.85 and +3.14) from the mean deviation score.

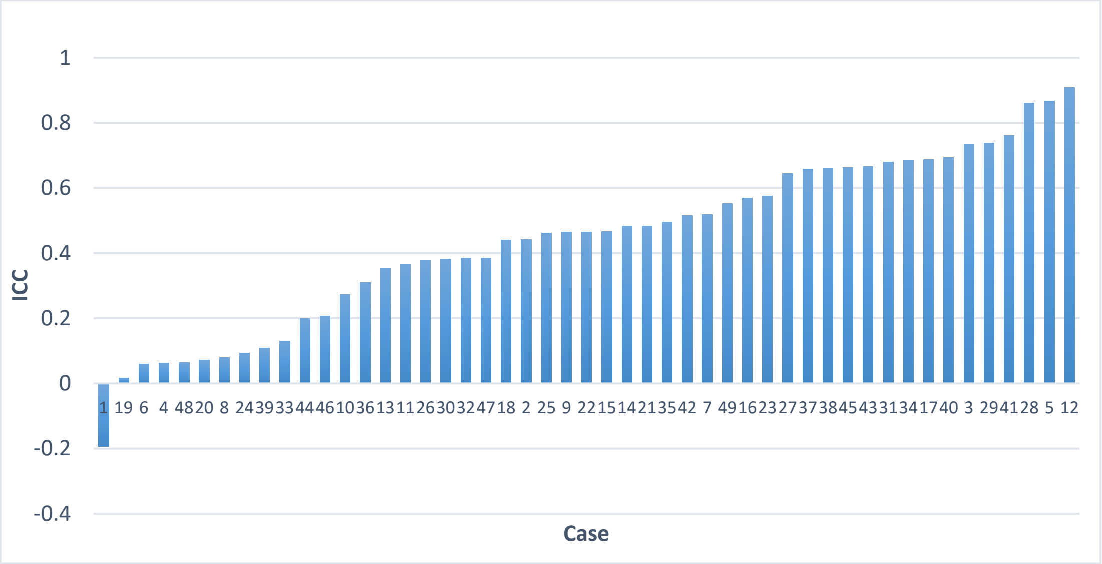

Figure S2. ICCs for each of the 49 cases in circulation 2. An additional measure of intra-case variability was provided by calculating an ICC for each of the 49 cases using the sTILs scores for the three FOV for the case as the variables for the ICC analysis. This generated a measure of agreement between the sTILs sores for the three FOV for the case. Cases for which the range of scores was similar for the three FOV had a high ICC (e.g., case 5 ICC 0.867; and case 41 ICC 0.762) regardless of whether the range of sTILs scores for the FOV was wide e.g., case 5 (range of scores for FOV 13, 14, 15 for case 5 seen in Supplementary Figure 3) or narrow e.g., case 41 (range of scores for FOV 121, 122, 123 for case 41 in Supplementary Figure 3). Thus, the case ICC gave a measure of variability between the FOV but was not a measure of accuracy.

(a)

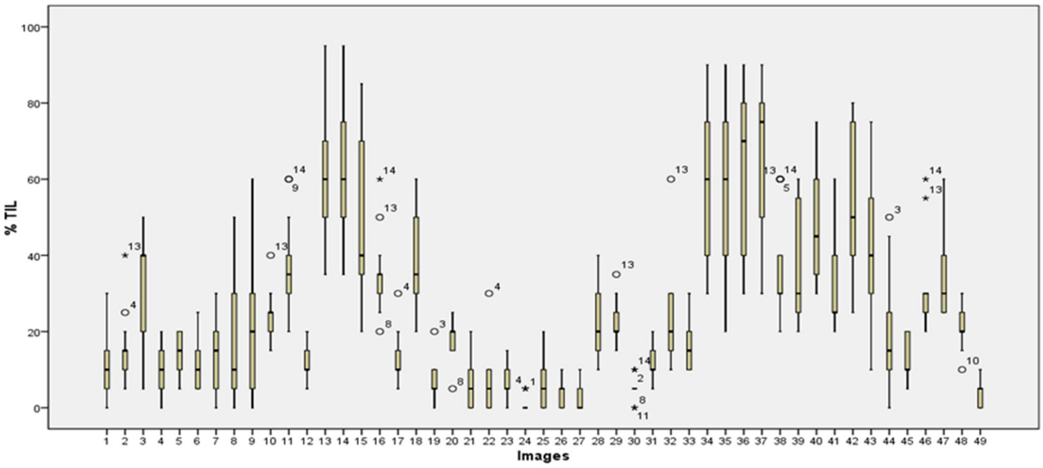

(b)

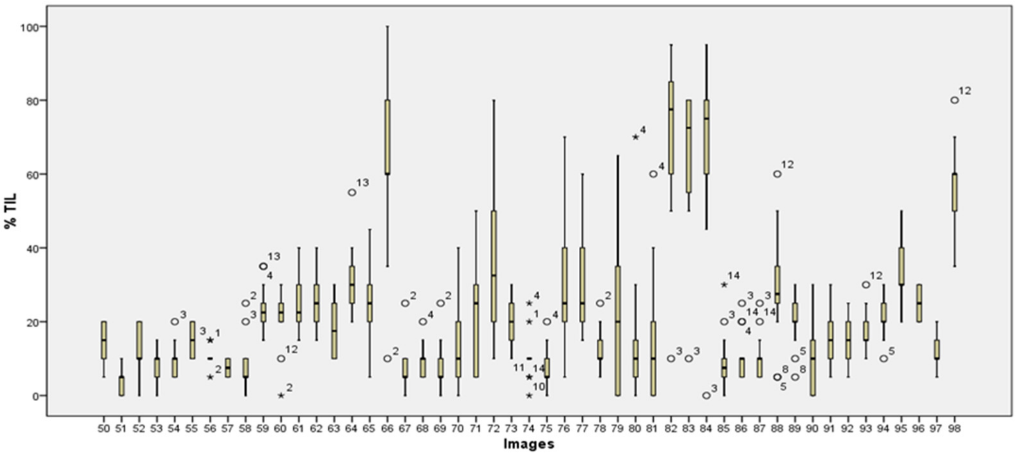

(c)

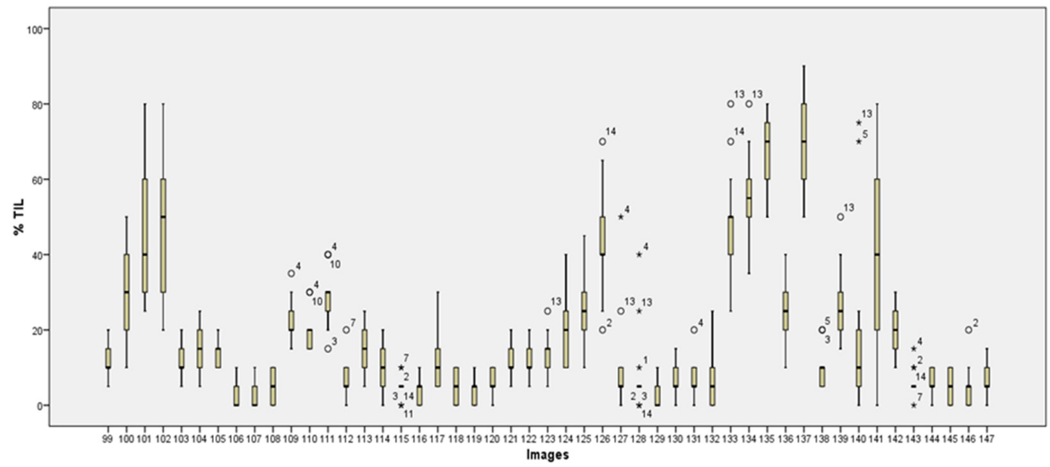

Figure S3. Distribution of sTILs scores for FOV (n = 147) in circulation 2. Legend. Box-and-Whisker plots of sTILs scores across the 147 FOV/images scored in circulation 2. The FOV/images, on the X-axis, are numbered consecutively from 1–49 (**a**); 50–98 (**b**); and 99–147 (**c**) with FOV/image 1,2,3 from case 1; FOV/image 4,5,6 from case 2 etc). The percentage sTILs score is given on the Y axis. The range of sTILs scores given was > 30% in 43 cases (29%).

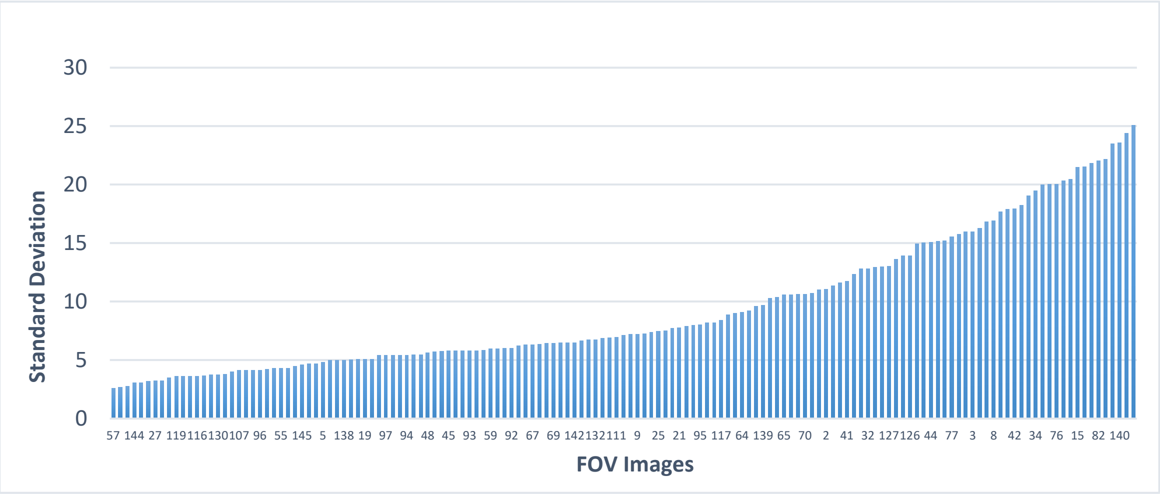

(a)

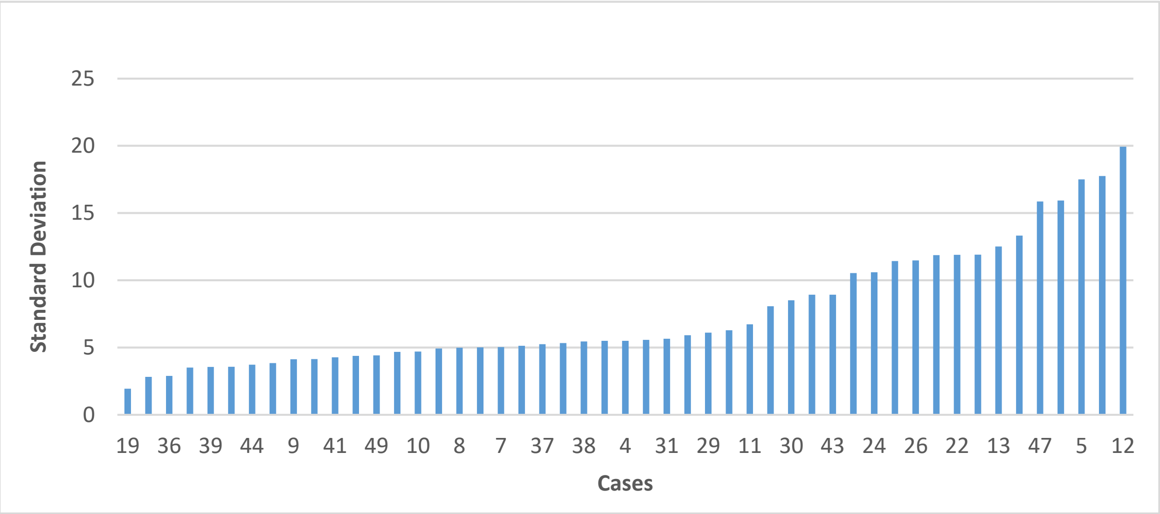

(b)

Figure S4. Standard deviation of sTILs scores for the FOV (n = 147) and cases (n = 49) in circulation 2. The standard deviation of sTILs scores was similar across (**a**) the 147 FOV/images (range 2.6 to 25) and (**b**) the 49 cases (range 1.9 to 19.9) in circulation 2.

Table S1. Clinico-pathological features for all cases (n = 49).

	**N (%)**
Age, years median (range)	56 (28–83)
Type	
NST	46 (94)
Other:	3 (6)
Grade	
1	0 (0)
2	7 (14)
3	39 (80)
No data	3 (6)
Chemotherapy	
NACT	4 (84)
Adjuvant	2 (4)
No treatment	3 (6)
No data	3 (6)
Post NACT	
pCR	19 (46)
non pCR	22 (54)
yp0	20 (49)
ypT1	14 (34)
ypT2	6 (15)
ypT3	1 (2)
yN0	33 (80)
yN1	6 (15)
yN2	0 (0)
yN3	2 (5)
Adjuvant or no treatment	
pT1	1 (20)
pT2	1 (20)
pT3	0 (0)
No data	3 (60)
N0	2 (40)
N1	0 (0)
N2	0 (0)
N3	0 (0)
No data	3 (60)
M0	43 (88)
M1	3 (6)
No data	3 (6)

Table S2. Inter-observer agreement between pathologists scoring sTILs across the FOV in circulation 2 (n = 147).

**sTILs**	**Circulation 2 (n = 14)**		
**(%)**	**ICC**	95% CI	p-value
	Absolute values		
	0.734	0.685–0.782	< 0.001
	Cutpoints		
≥ 10	0.431	0.370–0.500	< 0.001
≥ 20	0.566	0.505–0.631	< 0.001
≥ 25	0.602	0.542–0.664	< 0.001
≥ 30	0.595	0.535–0.657	< 0.001
≥ 40	0.603	0.544–0.665	< 0.001
≥ 50	0.581	0.520–0.644	< 0.001
≥ 60	0.467	0.405–0.536	< 0.001
